# Decomposition Analysis of Factors Affecting Changes in Industrial Wastewater Emission Intensity in China: Based on a SSBM-GMI Approach

**DOI:** 10.3390/ijerph15122779

**Published:** 2018-12-07

**Authors:** Yongyi Cheng, Liheng Lu, Tianyuan Shao, Manhong Shen, Laiqun Jin

**Affiliations:** 1 School of Business, Ningbo University, Ningbo 315211, China; styka1@163.com (T.S.); shenmanhong@nbu.edu.cn (M.S.); 2 Research Base of Ecological Civilization Construction, Ningbo University, Ningbo 315211, China; 3 School of Materials Science and Chemical Engineering, Ningbo University, Ningbo 315211, China; luliheng621@163.com

**Keywords:** industrial wastewater emission intensity, industrial economic growth, sustainable development, green total factor productivity, driving factor, production-theoretical decomposition analysis (PDA), slack-based measure (SBM)

## Abstract

This paper investigated the factors driving the changes in industrial wastewater emission intensity (IWEI) across provinces in China. To do this, we proposed a Super-efficiency Slacks-based Measure-Global Malmquist Index (SSBM-GMI) to decompose the change in IWEI into the effects from efficiency change (ECE), technological change (TCE), capital–wastewater substitution (KWE) and labor–wastewater substitution (LWE). The method was applied to conduct an empirical study using Chinese provincial data from 2003–2015. The main findings include the following: firstly, TCE was the dominant driving force behind the reduction in IWEI with an average annual contribution of −6.4% at the national level, followed by KWE (−5.3%), LWE (−1.8%) and ECE (1.2%). Secondly, significant differences exist in the driving factors behind the reduction in IWEI across regions. The reduction in IWEIs in the Northeast area and the Great Northwest area was mainly driven by productivity growth, while the reduction in IWEIs in the other areas was mainly driven by factor substitution. Thirdly, the shortage of KWE and LWE has impeded IWEI reduction in the Great Northwest area, the Middle Reaches of the Yellow River, the Northeast area and the North area. Finally, some particular policy implications were also recommended for reducing industrial wastewater emission in China.

## 1. Introduction

China, the world’s second largest economy, only ranked 120th among 180 countries and regions in the global Environmental Performance Index (EPI) in 2018 [1]. This ranking reflects the fact that China is still encountering great environmental challenges, particularly water pollution, which has become increasingly severe in China, and creating great impacts on the economy and overall quality of life [2,3]. According to the *China Environmental Quality Report* in 2016, out of the 31,000 km^2^ of total water surface comprised of 118 major lakes nationwide, only 23.7% of the assessed surface area had an annual water quality of Grade I-III. Moreover, of 6270 water functional areas evaluated across the country, only 58.7% met the water quality requirements [4]. Therefore, reducing water pollution in China, particularly within the industrial sector, which is a major contributor, has significant implications for achieving long-term environmental and economic sustainability [5].

**Figure 1:**
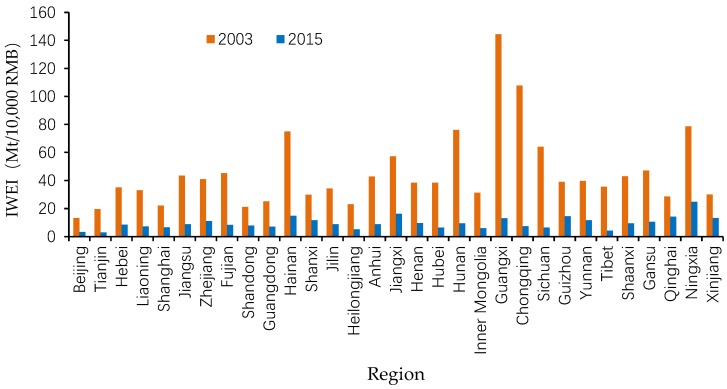
China’s regional industrial wastewater emission intensity, 2003–2015. Data resources: *Annual Statistic Report on Environment in China* [16]. IWEI: Industrial wastewater emission intensity.

To curb the deterioration of water environments, the Chinese government has enacted more than 130 policies relating to environmental protection since 1979 [6]. Furthermore, in an attempt to solve the water pollution problem once and for all, the Chinese government has recently formulated an ambitious action plan [7]. Regulating wastewater emission is the most direct means of limiting passive influences on the environment [8,9] since a significant part of waste generated by human activities ends up as wastewater [10]. Many researchers have investigated the driving forces behind the dynamics of China’s industrial wastewater emission, most of whom found that the emission intensity effect played a crucial role in the reduction of wastewater discharge in China [11,12,13,14,15]. However, there is still some research gaps in the cognition and research on the emission intensity effect because there is quite limited research that discusses the factors driving the change in industrial wastewater emission intensity (IWEI, the ratio between industrial wastewater and industrial output). As shown in Figure 1, IWEI varies greatly across the provinces of China, and it demonstrates a significantly decreasing trend in most provinces during 2003–2015. Nevertheless, what are the driving factors behind the dynamics of China’s regional IWEI? Are there substantial differences in the driving factors across regions? Until now, few scholars have conducted comprehensive studies on the above questions, even though such studies are of crucial significance not only for identifying the factors driving industrial wastewater emission, but to inform targeted pollution mitigation policies in China.

Based on the existing research, this paper makes a contribution in the following three ways. First, this study focuses on the factors driving the change in IWEI, which is relatively less well documented and understood compared to other indicators in the existing research. Second, this study proposes a Super-efficiency Slacks-based Measure-Global Malmquist Index (SSBM-GMI), which overcomes the drawbacks of the conventional green total factor productivity (TFP) index, and offers a more comprehensive approach to explaining the dynamics of IWEI by decomposing the changes in IWEI into the effects from efficiency change (ECE), technological change (TCE), capital-wastewater substitution (KWE) and labor-wastewater substitution (LWE). Third, this study performs an empirical study using Chinese provincial data, which helps to explain the driving factors behind the dynamics of China’s IWEI, and the significant regional differences.

The remainder of this paper is structured as follows. Section 2 presents the literature review. Section 3 introduces the methodology. Section 4 specifies the variables and data source, and reports the main results of the empirical study. Section 5 presents the discussion. Section 6 concludes with policy recommendations.

## 2. Literature Review

Following a series of pioneering studies employing the Environmental Kuznets Curve (EKC) [17,18,19], abundant research has focused on the relationship between economic growth and environmental pollution. Economic growth affects the level of environmental pollution in many ways. Without considering trade, the impacts mainly come from three factors: scale effect, structural effect, and technical effect [19]. Scale effect means that the expansion of economic output leads to increased resource consumption and increased pollution. Structural effect means that economic growth may cause industrial structural changes and lead to a shift in the share of resource-intensive (high-pollution) and knowledge-intensive (low-pollution) industries, resulting in the improvement or deterioration of environmental quality. Technical effect refers to the fact that economic growth may strengthen the capacity for technological innovation in economies, thereby increasing resource use efficiency and reducing the intensity of pollutant emissions, and consequently improving environmental quality. A large number of researchers have conducted empirical studies on these three effects [20,21,22]. Additionally, several studies have investigated the relationship between economic growth and water pollution in China; most of these have focused on measuring the “turning point” of water pollution by estimating the EKC [23,24,25]. Although these studies could be helpful for understanding the principles of water pollution change in China, they do not provide profound insights into the improvement of environment. 

Decomposition analysis is a useful method for identifying key driving factors behind sustainable development [26,27]. Studies on antecedent factors can help decision-makers develop more effective policies for controlling pollutants emissions, especially in situations where regional disparities exist [13]. Many researchers have analyzed the issue of industrial wastewater discharge in China by using the Index Decomposition Analysis (IDA) method [28]. For example, Lei et al. [11] used an additive version of the Logarithmic Mean Divisia Index (LMDI) decomposition method to examine the underlying driving forces behind the dynamics of China’s industrial wastewater. They found that the intensity effect exerted a major decremental effect on industrial water pollutant discharge. Additionally, Fujii et al. [12] used the LMDI method to calculate changes in wastewater pollutant emissions that resulted from cleaner production processes, end-of-pipe treatment, structural changes in industry, and changes in the scale of production from 1998 to 2010 in a number of Chinese industrial sectors. The study showed that COD emissions were mainly reduced through end-of-pipe treatments. Also using the LMDI method, Chen et al. [14] discussed the driving factors of wastewater discharge; the results revealed that the dominant factors affecting wastewater discharge are the economy and technological advance; a secondary factor is the efficiency of resource utilization, which brings about the unstable effect. Geng et al. [13] analyzed the spatial-temporal characteristics and driving forces of variations in industrial wastewater emission in China’s 31 provinces from 1995–2010. The results showed that technology improvement considerably offset emission increases during the study period. Jia et al. [15] decomposed changes in water pollutant discharge into three effects, namely, the economic output effect, the industrial structure effect, and the discharge intensity effect; the results indicated that the discharge intensity effect played a crucial role in the reduction of water pollutant discharge. The major limitation of these studies is that they are mainly based on a single factor, and do not consider other factors of production, e.g., capital, labor, etc. Consequently, they cannot provide in-depth information about the emission intensity effect, which has been identified as the dominant driving force in the reduction of wastewater emission. As pointed out in some of the literature, the existing IDA framework cannot investigate some of the potential driving factors affecting pollutant emissions, such as the substitution between pollutants and other production inputs, technical efficiency change and technological change [29,30,31]. 

To overcome this limitation, another commonly-used decomposition technique, PDA, which generally provides a better economic explanation for the decomposition of variables [32], could be an ideal supplement. By taking pollutants into account, TFP can be an effective indicator of the sustainability of industrial production technology. Many researchers have analyzed industrial water pollution under the total factor framework; most of them have focused on the calculation of the total factor efficiency of wastewater control while others have focused on measuring industrial green TFP incorporating wastewater with other pollutants. For instance, Chen and Fan [33] used the Data Envelopment Analysis (DEA) model to calculate the industrial wastewater control efficiency of various Chinese provinces. Based on a DEA meta-frontier model, Sala-Garrido et al. [34] compared the technical efficiency of different wastewater treatment systems. Liu et al. [35] adopted a DEA model that included undesirable outputs to assess the wastewater control efficiency of 10 industries in China between 2003 and 2012. Additionally, Yang and Li [36] calculated the total factor efficiency of wastewater control in different industrial sectors in China between 2003 and 2014. Fujii and Managi [37] used the weighted Russell directional distance model to evaluate the efficiency of industrial wastewater management in China between 2004 and 2014. Also using the DEA model, Li et al. [38] calculated green TFP for each industrial sector in China from 2001 to 2011 by estimating the Global Malmquist-Luenberger (GML) index using a Slacks-based Measure-Directional Distance Function (SBM-DDF). They found that a slightly greater portion of green TFP growth is attributable to technological progress (57%) rather than technical efficiency (43%). Additionally, Fujii et al. [39] calculated and decomposed productivity incorporating water pollutants in Chinese industrial sectors from 1992 to 2008 and found that the central and western regions have a trade-off relationship between economic and environmental performance. Chen et al. [40] employed directional distance function (DDF) and the GML productivity index to measure the green TFP growth of China’s 36 industrial sectors from 2000 to 2014. The results suggested that the growth of industrial economy sacrifices resources and environment to a certain degree. The above studies are helpful to understand the changes in wastewater control efficiency and green TFP in China’s industrial sector. Nevertheless, the detailed information about production technology was not used to conduct further decomposition analysis in these studies. Methodologically, an approach combining PDA and green TFP can be used to conduct a more in-depth investigation into potential driving factors affecting industrial wastewater emission, such as technological change, technical efficiency change and the substitution between wastewater and other production inputs. 

This study makes the following contributions to the extant literature: (1) it focuses on the factors driving the change in IWEI, which is relatively less well documented and understood compared to other indicators in the existing studies; (2) it decomposes the change in IWEI into ECE, TCE, KWE and LWE based on a novel combination of PDA and SSBM-GMI, which overcomes the drawbacks of the conventional green TFP index, and offers a more comprehensive approach to explain the dynamics of IWEI; and (3) it performs an empirical study using Chinese provincial data, which helps to explain the driving factors behind the dynamics of China’s IWEI, and the significant regional differences.

## 3. Methodology

### 3.1. The Initial Decompsition Framework

We decomposed IWEI change into multiple components using non-radial distance functions. This study adopts the nonparametric Data Envelopment Analysis (DEA) piecewise linear production frontiers, following Färe et al. [41]. Non-radial distance function is used to measure efficiency gap between production frontier and a certain specific decision-making unit (DMU, that is the provinces in this study). To ensure that the decomposition is simple and intuitive, pollutant is treated as input in this study (please see Appendix A for a detailed explanation). Accordingly, we take capital (*K*), labor (*L*) and wastewater (*W*) as inputs, and gross output (*Y*) as the output. Hence, for each time period (*t* = 1, 2, …, T), a production possibility set (PPS) can be given by the following:

(1) $$PPS^{t}\;=\;\left\{\left(K^{t},L^{t},W^{t},Y^{t}\right)|\left(K^{t},L^{t},W^{t}\right)\;\mathrm{can}\;\mathrm{produce}\;Y^{t}\right\}$$

In Equation (1), the production technology is assumed to follow all the standard axioms of production theory, including the assumptions of bounded set, bounded convexity, etc. [42,43]. Thus, the input-oriented non-radial distance function can be defined as follows:

(2) $$D^{t}\left(K^{t},L^{t},W^{t},Y^{t}\right)\;=\;\inf\;\left\{\theta =\frac{1}{3}\left(\theta_{K}\;+\;\theta_{L}\;+\;\theta_{W}\right):\left(\theta_{K}K^{t},\theta_{L}L^{t},\theta_{W}W^{t},Y^{t}\right)\in PPS^{t}\right\}$$

In Equation (2), capital, labor and wastewater emission are minimized, given that output is unchanged. The specific DMU is inefficient, if the value of $D^{t}\left(K^{t},L^{t},W^{t},Y^{t}\right)$ is lower than 1. By contrast, the DMU is efficient, given that the values of $D^{t}\left(K^{t},L^{t},W^{t},Y^{t}\right)$ is equal to 1. Therefore, based on the technology in time period *t* as a reference, and with the assumption of constant returns to scale (CRS), the change in IWEI between time period *t* and *t* + 1 can be written as follows:

(3) $$\begin{array}{cc} \frac{IWEI^{t\;+\;1}}{IWEI^{t}}\;= & \frac{W^{t\;+\;1}/Y^{t\;+\;1}}{W^{t}/Y^{t}}\\ & \;\;=\;\frac{W^{t\;+\;1}/\left(Y^{t\;+\;1}/D^{t}\left(K^{t\;+\;1},L^{t\;+\;1},W^{t\;+\;1},Y^{t\;+\;1}\right)\right)}{W^{t}/\left(Y^{t}/D^{t}\left(K^{t},L^{t},W^{t},Y^{t}\right)\right)}\\ & \;\;\times \;\frac{D^{t}\left(K^{t},L^{t},W^{t},Y^{t}\right)}{D^{t}\left(K^{t\;+\;1},L^{t\;+\;1},W^{t\;+\;1},Y^{t\;+\;1}\right)}\\ & \;\;=\frac{D^{t}\left(K^{t},L^{t},W^{t},Y^{t}\right)}{D^{t\;+\;1}\left(K^{t\;+\;1},L^{t\;+\;1},W^{t\;+\;1},Y^{t\;+\;1}\right)}\;\\ & \;\;\times \;\frac{D^{t\;+\;1}\left(K^{t\;+\;1},L^{t\;+\;1},W^{t\;+\;1},Y^{t\;+\;1}\right)}{D^{t}\left(K^{t\;+\;1},L^{t\;+\;1},W^{t\;+\;1},Y^{t\;+\;1}\right)}\\ & \;\;\times \;\frac{W^{t\;+\;1}/\left(Y^{t\;+\;1}/D^{t}\left(K^{t\;+\;1},L^{t\;+\;1},W^{t\;+\;1},Y^{t\;+\;1}\right)\right)}{W^{t}/\left(Y^{t}/D^{t}\left(K^{t},L^{t},W^{t},Y^{t}\right)\right)} \end{array}$$

which suggests that the change in *IWEI* can be decomposed into three components. The first measures the change in distance of observed DMU from the production frontier between the two periods, i.e., the reciprocal of efficiency change (EC). The second measures the shift in production frontier between the two periods, i.e., the reciprocal of technological change (TC). And the third measures the change in minimum potential wastewater emission intensity between the two periods, using the technical level in time period *t* as a reference. 

### 3.2. The Improved Decompsition Framework

As shown in various studies, the conventional green TFP index has some drawbacks—infeasible solutions may be encountered and the index is not circular, etc., which are referred to as the “discriminating power problem” and “technical regress” [32,44]. To overcome these drawbacks, many improved methods have been being developed. Tone [45,46] proposed the super-efficiency model based on the non-radial, non-angular Slacks-based Measure (SBM) model to address the “discriminating power problem”. Pastor and Lovell [47] proposed a new index based on a global benchmark technology. Oh [48] further developed it with sequential technology, which is circular and can address “technical regress”, and this has been widely used in the studies of productivity growth in recent years [49]. Thus, to ensure the decomposition results will be accurate, we combined global frontier technology with sequential frontier technology, and use the super-efficiency SBM model to avoid “discriminating power problem” and “technical regress”. The PPS of the sequential frontier at time *t* is given by

(4) $$PPS^{Seq\left(t\right)}\;=\;\left\{PPS^{1}\cup PPS^{2}\cup \ldots \cup PPS^{t}\right\}$$

Also, the PPS of the global frontier is given by

(5) $$PPS^{Glb}\;=\;\left\{PPS^{1}\cup PPS^{2}\cup \ldots \cup PPS^{T}\right\}$$

where $PPS^{Seq\left(t\right)}$ and $PPS^{Glb}$ denote the specific technologies of the sequential frontier and global frontier (i.e., best practice frontier), respectively. The superscript *Seq* and *Glb* denote the sequential frontier technology and the global frontier technology, respectively. Technically, the distance functions used in this study are defined as follows.

(6) $$D^{Glb}\left(K^{t},L^{t},W^{t},Y^{t}\right)\;=\;\inf\;\left\{\theta \;=\;\frac{1}{3}\left(\theta_{K}\;+\;\theta_{L}\;+\;\theta_{W}\right):\left(\theta_{K}K^{t},\theta_{L}L^{t},\theta_{W}W^{t},Y^{t}\right)\in PPS^{Glb}\right\}$$

(7) $$D^{Seq\left(t\right)}\left(K^{t},L^{t},W^{t},Y^{t}\right)\;=\;\inf\;\left\{\theta \;\;=\;\frac{1}{3}\left(\theta_{K}\;+\;\theta_{L}\;+\;\theta_{W}\right):\left(\theta_{K}K^{t},\theta_{L}L^{t},\theta_{W}W^{t},Y^{t}\right)\in PPS^{Seq\left(t\right)}\right\}$$

The specific DMU is globally or sequentially inefficient, if the value of $D^{Glb}\left(K^{t},L^{t},W^{t},Y^{t}\right)$ or $D^{Seq\left(t\right)}\left(K^{t},L^{t},W^{t},Y^{t}\right)$ is lower than 1. By contrast, the DMU is most efficient, given that the values of $D^{Glb}\left(K^{t},L^{t},W^{t},Y^{t}\right)$ and $D^{Seq\left(t\right)}\left(K^{t},L^{t},W^{t},Y^{t}\right)$ are both equal to 1. Therefore, based on the technologies at the sequential frontier and global frontier as references, the change in IWEI between time period *t* and *t* + 1 can be rewritten as follows:

(8) $$\begin{array}{c} \frac{IWEI^{t\;+\;1}}{IWEI^{t}}\;=\;\frac{W^{t\;+\;1}/Y^{t\;+\;1}}{W^{t}/Y^{t}}\\ =\;\frac{W^{t\;+\;1}/\left(Y^{t\;+\;1}/D^{Glb}\left(K^{t\;+\;1},L^{t\;+\;1},W^{t\;+\;1},Y^{t\;+\;1}\right)\right)}{W^{t}/\left(Y^{t}/D^{Glb}\left(K^{t},L^{t},W^{t},Y^{t}\right)\right)}\\ \times \;\frac{D^{Glb}\left(K^{t},L^{t},W^{t},Y^{t}\right)}{D^{Glb}\left(K^{t\;+\;1},L^{t\;+\;1},W^{t\;+\;1},Y^{t\;+\;1}\right)}\\ =\;\frac{D^{Seq\left(t\right)}\left(K^{t},L^{t},W^{t},Y^{t}\right)}{D^{Seq\left(t\;+\;1\right)}\left(K^{t\;+\;1},L^{t\;+\;1},W^{t\;+\;1},Y^{t\;+\;1}\right)}\\ \times \;\frac{D^{Glb}\left(K^{t},L^{t},W^{t},Y^{t}\right)/D^{Seq\left(t\right)}\left(K^{t},L^{t},W^{t},Y^{t}\right)}{D^{Glb}\left(K^{t\;+\;1},L^{t\;+\;1},W^{t\;+\;1},Y^{t\;+\;1}\right)/D^{Seq\left(t\;+\;1\right)}\left(K^{t\;+\;1},L^{t\;+\;1},W^{t\;+\;1},Y^{t\;+\;1}\right)}\\ \times \;\frac{W^{t\;+\;1}/\left(Y^{t\;+\;1}/D^{Glb}\left(K^{t\;+\;1},L^{t\;+\;1},W^{t\;+\;1},Y^{t\;+\;1}\right)\right)}{W^{t}/\left(Y^{t}/D^{Glb}\left(K^{t},L^{t},W^{t},Y^{t}\right)\right)} \end{array}$$

which suggests that the change in *IWEI* can be decomposed into three components. The first measures the change in distance of observed DMU from the sequential frontier between the two periods, i.e., the reciprocal of EC. The second measures the change in distance of the sequential frontier from the global frontier between the two periods, i.e., the reciprocal of TC. And the third measures the change in minimum potential wastewater emission intensity between the two periods, using the technical level at the global frontier as a reference. According to these above definitions, and with the assumption of CRS, it can be easily verified that the input distance function is homogeneous with degree −1 in inputs: $D^{Glb}\left(\beta K^{t},\beta L^{t},\beta W^{t},Y^{t}\right)=\beta^{-1}D^{Glb}\left(K^{t},L^{t},W^{t},Y^{t}\right)$, where β is a positive scalar [50,51]. Thus, the third term in the bottom line of Equation (8) can be further decomposed as follows:

(9) $$\frac{W^{t\;+\;1}/\left(Y^{t\;+\;1}/D^{Glb}\left(K^{t\;+\;1},L^{t\;+\;1},W^{t\;+\;1},Y^{t\;+\;1}\right)\right)}{W^{t}/\left(Y^{t}/D^{Glb}\left(K^{t},L^{t},W^{t},Y^{t}\right)\right)}\;=\;\frac{D^{Glb}\left(k^{t\;+\;1},l^{t\;+\;1},1,Y^{t\;+\;1}\right)/Y^{t\;+\;1}}{D^{Glb}\left(k^{t},l^{t},1,Y^{t}\right)/Y^{t}}$$

where $k^{t}=K^{t}/W^{t}$ and $l^{t}=L^{t}/W^{t}$ denote the capital-wastewater and labor-wastewater ratio respectively. Additionally, given the assumption of CRS, which implies that the input distance function is homogeneous with degree +1 in outputs: $D^{Glb}\left(K^{t},L^{t},W^{t},\alpha Y^{t}\right)=\alpha D^{Glb}\left(K^{t},L^{t},W^{t},Y^{t}\right)$, where α is a positive scalar, then Equation (9) can be rewritten as follows:

(10) $$\frac{W^{t\;+\;1}/\left(Y^{t\;+\;1}/D^{Glb}\left(K^{t\;+\;1},L^{t\;+\;1},W^{t\;+\;1},Y^{t\;+\;1}\right)\right)}{W^{t}/\left(Y^{t}/D^{Glb}\left(K^{t},L^{t},W^{t},Y^{t}\right)\right)}\;=\;\frac{D^{Glb}\left(k^{t\;+\;1},l^{t\;+\;1},1,1\right)}{D^{Glb}\left(k^{t},l^{t},1,1\right)}$$

Thus, Equation (8) can be rewritten as:

(11) $$\begin{array}{c} \frac{IWEI^{t\;+\;1}}{IWEI^{t}}\\ =\;\frac{D^{Seq\left(t\right)}\left(K^{t},L^{t},W^{t},Y^{t}\right)}{D^{Seq\left(t\;+\;1\right)}\left(K^{t\;+\;1},L^{t\;+\;1},W^{t\;+\;1},Y^{t\;+\;1}\right)}\\ \times \;\frac{D^{Glb}\left(K^{t},L^{t},W^{t},Y^{t}\right)/D^{Seq\left(t\right)}\left(K^{t},L^{t},W^{t},Y^{t}\right)}{D^{\mathrm{Glb}}\left(K^{t\;+\;1},L^{t\;+\;1},W^{t\;+\;1},Y^{t\;+\;1}\right)/D^{Seq\left(t\;+\;1\right)}\left(K^{t\;+\;1},L^{t\;+\;1},W^{t\;+\;1},Y^{t\;+\;1}\right)}\\ \times \;\frac{D^{Glb}\left(k^{t},l^{t},1,1\right)}{D^{Glb}\left(k^{t-1},l^{t-1},1,1\right)}\;=\;\frac{1}{EC^{t,t\;+\;1}}\;\times \;\frac{1}{TC^{t,t\;+\;1}}\;\times \;\frac{D^{Glb}\left(k^{t\;+\;1},l^{t\;+\;1},1,1\right)}{D^{Glb}\left(k^{t},l^{t},1,1\right)} \end{array}$$

which suggests that the change in *IWEI* can be decomposed into three components: the first measures the effect of EC; the second measures the effect of TC; and the third depends on the changes in *k* (capital–wastewater ratio) and *l* (labor–wastewater ratio). To investigate the effects of the changes in *k* and *l* between times *t* − 1 and *t*, we follow the method of Ang et al. [52] and Wang [51]. The third term in the bottom line of Equation (11) can be further decomposed as follows:

(12) $$\begin{array}{cc} \frac{D^{Glb}\left(k^{t\;+\;1},l^{t\;+\;1},1,1\right)}{D^{Glb}\left(k^{t},l^{t},1,1\right)} & \\ & =\;{\left[\frac{D^{Glb}\left(k^{t\;+\;1},l^{t},1,1\right)}{D^{Glb}\left(k^{t},l^{t},1,1\right)}\;\times \;\frac{D^{Glb}\left(k^{t\;+\;1},l^{t\;+\;1},1,1\right)}{D^{Glb}\left(k^{t},l^{t\;+\;1},1,1\right)}\right]}^{\frac{1}{2}}\\ & \times \;{\left[\frac{D^{Glb}\left(k^{t},l^{t\;+\;1},1,1\right)}{D^{Glb}\left(k^{t},l^{t},1,1\right)}\;\times \;\frac{D^{Glb}\left(k^{t\;+\;1},l^{t\;+\;1},1,1\right)}{D^{Glb}\left(k^{t\;+\;1},l^{t},1,1\right)}\right]}^{\frac{1}{2}} \end{array}$$

Therefore, based on the technologies at the sequential frontier and global frontier as references, the change in IWEI between time period *t* and *t* + 1 can be decomposed as:

(13) $$\begin{array}{cc} \frac{IWEI^{t\;+\;1}}{IWEI^{t}}\;= & \frac{1}{EC^{t,t\;+\;1}}\;\times \;\frac{1}{TC^{t,t\;+\;1}}\\ & \;\;\times \{{\left[\frac{D^{Glb}\left(k^{t\;+\;1},l^{t},1,1\right)}{D^{Glb}\left(k^{t},l^{t},1,1\right)}\;\times \;\frac{D^{Glb}\left(k^{t\;+\;1},l^{t\;+\;1},1,1\right)}{D^{Glb}\left(k^{t},l^{t\;+\;1},1,1\right)}\right]}^{\frac{1}{2}}\\ & \;\;\times {\left[\frac{D^{Glb}\left(k^{t},l^{t\;+\;1},1,1\right)}{D^{Glb}\left(k^{t},l^{t},1,1\right)}\;\times \;\frac{D^{Glb}\left(k^{t\;+\;1},l^{t\;+\;1},1,1\right)}{D^{Glb}\left(k^{t\;+\;1},l^{t},1,1\right)}\right]}^{\frac{1}{2}}\}\\ & \;\;=\;\left(ECE^{t,t\;+\;1}\times TCE^{t,t\;+\;1}\right)\\ & \;\;\times \;\left(KWE^{t,t\;+\;1}\times LWE^{t,t\;+\;1}\right)\;=PE^{t,t\;+\;1}\times FSE^{t,t\;+\;1} \end{array}$$

which suggests that the change in *IWEI* can be decomposed into four components. The first component, *ECE^t,t^*^+1^, measures the effect of technical efficiency change; values smaller than 1 indicate that improvements in technical efficiency have promoted the decrease of *IWEI*. The second component, *TCE^t,t^*^+1^, measures the effect of technological change; values smaller than 1 indicate that technological advancements have contributed to the decline of *IWEI*. The third component, *KWE^t,t^*^+1^, denotes the effect of capital–wastewater substitution, which measures the effects on the change in *IWEI* of changes in the capital–wastewater ratio; values smaller than 1 indicate that capital–wastewater substitution has contributed to the decline of *IWEI*. The fourth component, *LWE^t,t^*^+1^, denotes the effect of capital–wastewater substitution, which measures the effects on the change in *IWEI* of changes in the labor–wastewater ratio. In this case, values smaller than 1 also signify that labor–wastewater substitutions have promoted the decline of *IWEI*. 

The concept of the Factor Substitution Effect (FSE) has been widely used to study the substitution effect between energy and other factors [53,54]. Theoretically, whether the expansion of the scale of industrial production reduces or increases the intensity of pollutant emissions depends on how productivity changes, and how new inputs are allocated between different production processes and industries, e.g., pollution-reducing process or pollution-generating process, lightly polluting industry or heavily polluting industry substitute capital for pollution or substitute pollution for capital. Therefore, the first two components, *ECE^t,t^*^+1^ and *TCE^t,t^*^+1^, measure the productivity effect (PE) while the last two components, *KWE^t,t^*^+1^ and *LWE^t,t^*^+1^, measure the substitution effect between wastewater and the other two production factors, i.e., the FSE. 

### 3.3. The Estimation Model

To calculate the super-efficiency of a specific DMU, $PPS^{Seq}$ and $PPS^{Glb}$ are constructed by eliminating the observations of that specific DMU. Hence, the distance function $D\left(K_{0},\;L_{0},\;W_{0},\;Y_{0}\right)$, which measures the distance of observed DMU*_0_* from the frontier, can be calculated by solving the linear programming (LP) problem given below:

(14) $$D\left(K_{0},\;L_{0},\;W_{0},\;Y_{0}\right)=\min\;\left\{\theta =\frac{1}{3}\left(\frac{K_{0}-s^{K}}{K_{0}}\;+\;\frac{L_{0}-s^{L}}{L_{0}}\;+\;\frac{W_{0}-s^{W}}{W_{0}}\right)\right\}\;s.\;t.\;\{\begin{array}{c} K_{0}=\sum_{i\neq 0}\left(K\lambda \right)\;+\;s^{K}\\ L_{0}=\sum_{i\neq 0}\left(L\lambda \right)\;+\;s^{L}\\ W_{0}=\sum_{i\neq 0}\left(W\lambda \right)\;+\;s^{W}\\ Y_{0}\leq \sum_{i\neq 0}\left(Y\lambda \right)\\ \lambda \geq 0,s^{K}\geq 0,s^{L}\geq 0,s^{W}\geq 0 \end{array}$$

where λ denotes the weight matrix, and $s^{K}$, $s^{L}$ and $s^{W}$ denote the slack in capital investment, labor investment and wastewater emission, respectively.

Our decomposition method combined the super-efficiency SBM model, global frontier technology, sequential frontier technology and the Malmquist index, which was named the SSBM-GMI method.

## 4. Data and Empirical Results

### 4.1. Data

To achieve statistical consistency, our research included 31 regions of mainland China (i.e., excluding Hong Kong, Macao, and Taiwan), and the study period covered the years 2003–2015. The data were collected from the *China Statistical Yearbook* [55], the *China Statistical Yearbook on Environment* [56], and the *Annual Statistical Report on Environment in China* [16]. All monetary variables are adjusted to the 2001 constant price using the corresponding price indices. Capital input (*K*): capital stock is estimated from annual fixed assets investment in each year by adopting the Perpetual Inventory Method [57]. Labor input (*L*): the average number of workers each year in the industrial sector. Wastewater (*W*): the amount of industrial wastewater emission. Output (*Y*): the industrial value added is defined as industrial output of each province. Table 1 lists the descriptive statistics for the relevant variables. 

**Figure 2:**
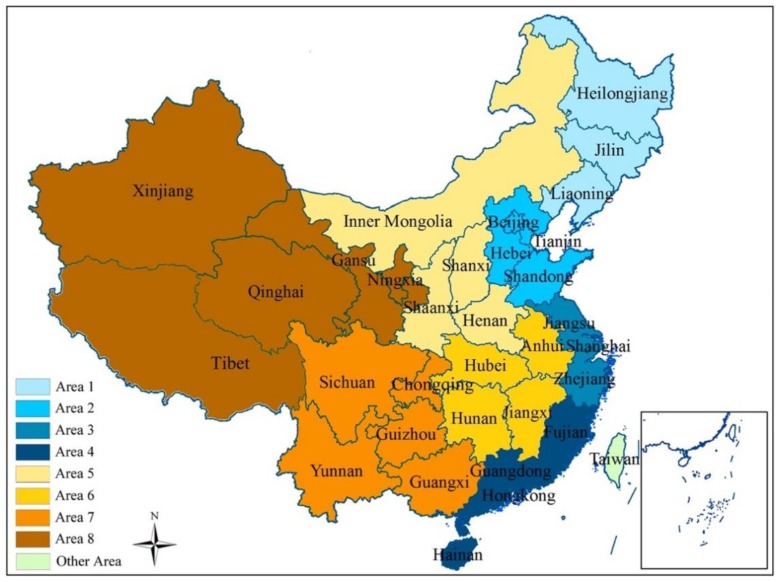
The eight study areas in China.

To obtain a thorough understanding of the differences between the Chinese provinces, this study applied criteria from the “Development Strategy and Regional Economic Research Section of Development Research Center of China’s State Council” to divide the 31 provinces of China into eight geographic study areas: the Northeast area (Area 1), the North area (Area 2), the Eastern Coastal area (Area 3), the Southern Coastal area (Area 4), the Middle Reaches of the Yellow River (Area 5), the Middle Reaches of the Yangtze River (Area 6), the Southwest area (Area 7), and the Great Northwest area (Area 8) [32]. Figure 2 shows the provinces in each area.

### 4.2. Regional Difference in IWEI Change

The IWEI in most provinces declined dramatically between 2003 and 2015, with an average decline of 75.98%, as can be seen in Table 2. Specifically, Guangxi province experienced the sharpest decline, 91%, while Qinghai province only achieved a decline of 50.31%, the lowest. A total of 15 provinces performed at below the average level, namely Beijing, Hebei, Shanxi, Jilin, Shanghai, Zhejiang, Jiangxi, Shandong, Henan, Guangdong, Guizhou, Yunnan, Qinghai, Ningxia, and Xinjiang. The IWEIs vary greatly among regions. Guangxi province had the highest IWEI in 2003, which is more than ten times Beijing’s IWEI, the lowest one while Ningxia province had the highest IWEI in 2015, which is almost nine times Tianjin’s IWEI, the lowest one. 

**Figure 3:**
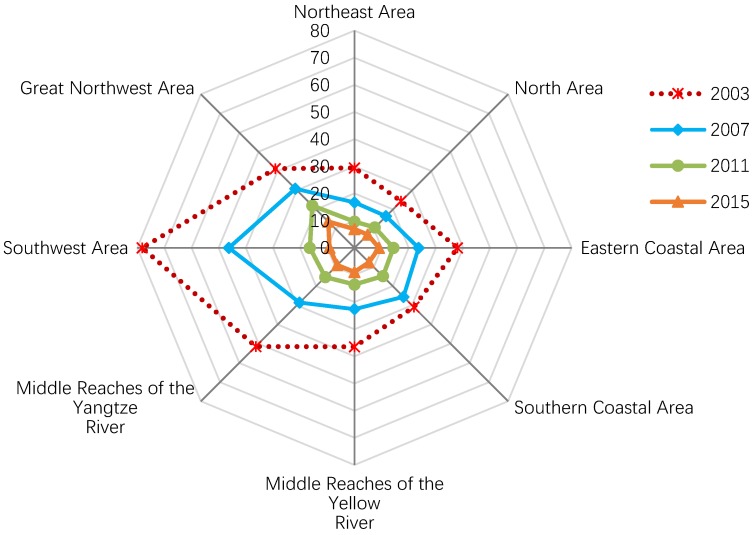
Regional contrast of the change in industrial wastewater emission intensity, 2003–2015. Data resources: *Annual Statistic Report on Environment in China* [16].

To further investigate the regional differences, the IWEIs in eight areas were calculated, and a variation trend was constructed, as shown in Figure 3. In 2003, the Southwest area had the highest IWEI, followed by the Middle Reaches of the Yangtze River and the Great Northwest area, while the North area had the lowest IWEI, followed by the Northeast area and the Southern Coastal area. Between 2003 and 2015, the IWEIs of eight areas all decreased distinctly, and the Southwest area experienced an especially sharp decline, followed by the Middle Reaches of the Yangtze River. The difference between regions reduced greatly during the study period. However, at the end of the study period, there remained a significant gap between the Great Northwest area and the other areas. 

### 4.3. Decomposition Results of Factors Affecting IWEI Change

Based on the results in Section 3, the changes in IWEI of each province between 2003 and 2015 were decomposed using the SSBM-GMI approach; the results are reported in Table 3. Column 1 of Table 3 reports the annual change in IWEI for each province, and Columns 2–7 report the annual average contributions to the change in IWEI for each province from ECE, TCE, PE, KWE, LWE, and FSE.

#### 4.3.1. Results at the Regional Level 

As shown in column 2 of Table 3, the annual average ECE value is bigger than 1 for most regions, indicating that technical efficiency change played a negative role in the decrease in IWEI. Exceptions to this observation include only ten regions: Liaoning, Tianjin, Hainan, Inner Mongolia, Hubei, Guangxi, Chongqing, Sichuan, and Gansu. Column 3 of Table 3 indicates that technological change plays a positive role in the decrease in IWEI in all provinces. As shown in column 4, the PE values are significantly below 1 for all provinces, indicating that productivity growth contributed significantly to the decline in their IWEI. Additionally, when ECE and TCE (the two sub-items of the PE) are compared, it can be seen that, in all provinces, technological advances have played a more important role than efficiency improvement in the decline in their IWEI. 

Column 5 of Table 3 indicates that the KWE plays a positive role in the decrease in IWEI in all regions; column 6 indicates that the LWE also plays a positive role in the decrease in IWEI, except in certain regions, including Shandong, Shanxi, Guizhou, Tibet, Gansu, Qinghai, and Ningxia, which are mostly in Midwest China. As is shown in column 7 of Table 3, the annual average values of the FSE are smaller than 1 for all provinces, indicating that the factor substitution has promoted the reduction of China’s provincial IWEI. 

Among the four components ECE, TCE, KWE, and LWE, TCE contributes the most to IWEI reduction in 20 of the 31 regions. KWE contributed the most to IWEI reduction in the remaining 11 regions—which include Hebei, Jiangsu, Zhejiang, Hainan, Henan, Anhui, Hunan, Guangxi, Chongqing, Sichuan and Ningxia—suggesting that the reduction in IWEI in these regions is strongly driven by capital–wastewater substitution. 

**Figure B1:**
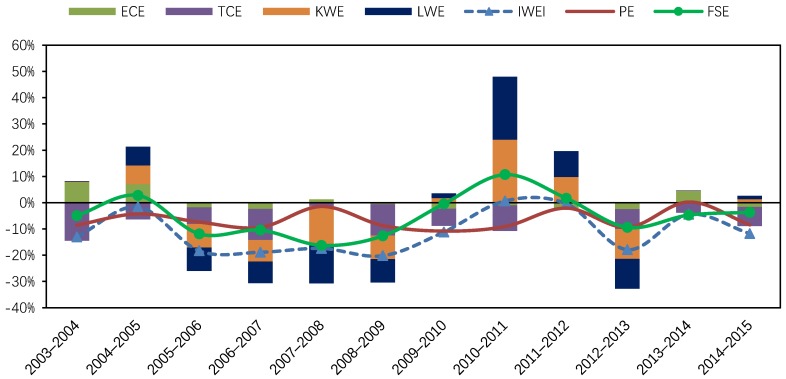
Driving factors of industrial wastewater emission intensity change in the Northeast Area, 2003–2015. ECE: the effect of technical efficiency change; TCE: the effect of technological change; KWE: the effect of capital–wastewater substitution; LWE: the effect of labor–wastewater substitution; IWEI: industrial wastewater emission intensity; PE: productivity effect; FSE: factor substitution effect.

**Figure B2:**
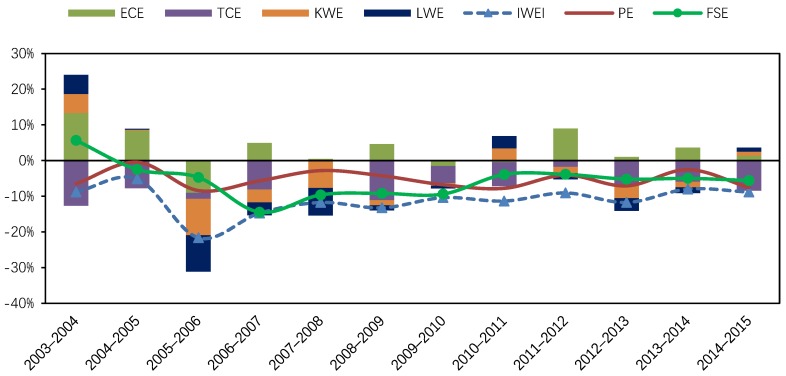
Driving factors of industrial wastewater emission intensity change in the North Area, 2003–2015. ECE: the effect of technical efficiency change; TCE: the effect of technological change; KWE: the effect of capital–wastewater substitution; LWE: the effect from labor–wastewater substitution; IWEI: industrial wastewater emission intensity; PE: productivity effect; FSE: factor substitution effect.

**Figure B3:**
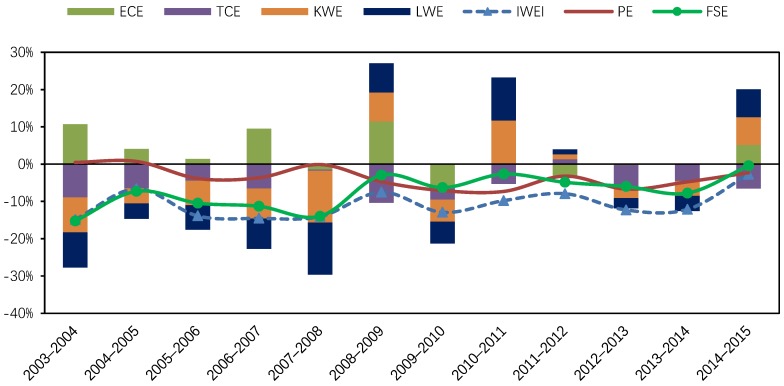
Driving factors of industrial wastewater emission intensity change in the Eastern Coastal Area, 2003–2015. ECE: the effect of technical efficiency change; TCE: the effect of technological change; KWE: the effect of capital–wastewater substitution; LWE: the effect of labor–wastewater substitution; IWEI: industrial wastewater emission intensity; PE: productivity effect; FSE: factor substitution effect.

**Figure B4:**
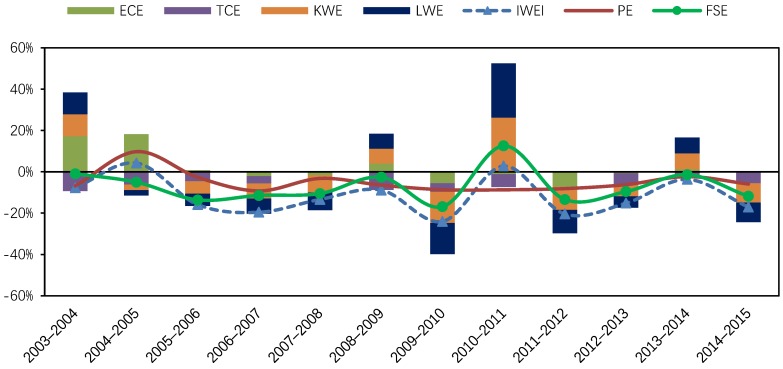
Driving factors of industrial wastewater emission intensity change in the Southern Coastal Area, 2003–2015. ECE: the effect of technical efficiency change; TCE: the effect of technological change; KWE: the effect of capital–wastewater substitution; LWE: the effect of labor–wastewater substitution; IWEI: industrial wastewater emission intensity; PE: productivity effect; FSE: factor substitution effect.

**Figure B5:**
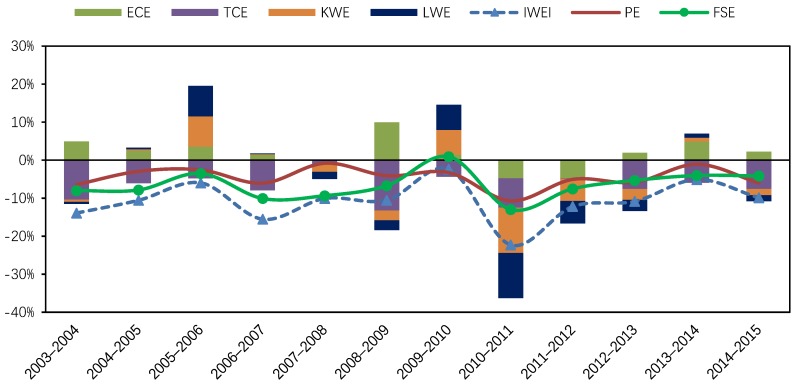
Driving factors of industrial wastewater emission intensity change in the Middle Reaches of the Yellow River, 2003–2015. ECE: the effect of technical efficiency change; TCE: the effect of technological change; KWE: the effect of capital–wastewater substitution; LWE: the effect of labor–wastewater substitution; IWEI: industrial wastewater emission intensity; PE: productivity effect; FSE: factor substitution effect.

**Figure B6:**
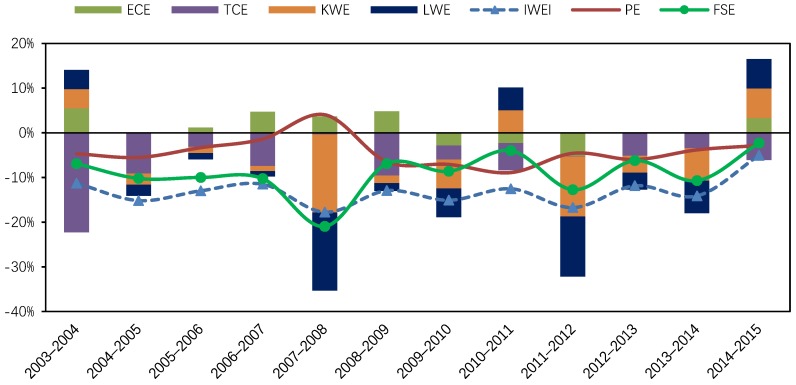
Driving factors of industrial wastewater emission intensity change in the Middle Reaches of the Yangtze River, 2003–2015. ECE: the effect of technical efficiency change; TCE: the effect of technological change; KWE: the effect of capital–wastewater substitution; LWE: the effect of labor–wastewater substitution; IWEI: industrial wastewater emission intensity; PE: productivity effect; FSE: factor substitution effect.

**Figure B7:**
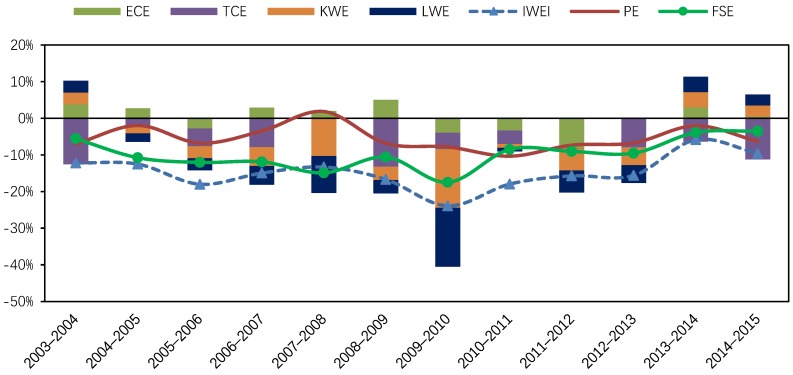
Driving factors of industrial wastewater emission intensity change in the Southwest Area, 2003–2015. ECE: the effect of technical efficiency change; TCE: the effect of technological change; KWE: the effect of capital–wastewater substitution; LWE: the effect of labor–wastewater substitution; IWEI: industrial wastewater emission intensity; PE: productivity effect; FSE: factor substitution effect.

**Figure B8:**
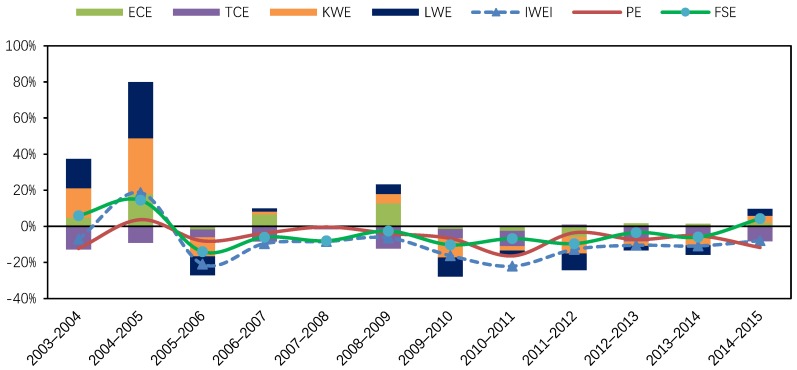
Driving factors of industrial wastewater emission intensity change in the Great Northwest Area, 2003–2015. ECE: the effect from technical efficiency change; TCE: the effect of technological change; KWE: the effect of capital–wastewater substitution; LWE: the effect of labor–wastewater substitution; IWEI: industrial wastewater emission intensity; PE: productivity effect; FSE: factor substitution effect.

When comparing the changes in IWEI in eight areas, it can be seen that the Southwest area experienced the quickest annual reduction, −14.8%, followed by the Middle Reaches of the Yangtze River while the Great Northwest area experienced the slowest annual reduction, −10%, followed by the Middle Reaches of the Yellow River. Furthermore, among the four components ECE, TCE, KWE, and LWE, TCE contributes the most to IWEI reduction in six of the eight areas, and KWE contributed the most to IWEI reduction in the remaining two areas, the Southwest area and the Middle Reaches of the Yangtze River, suggesting that the reduction in IWEI in these two areas is strongly driven by capital substitution. The decomposition results across the eight areas over the study period are provided in Figure B1, Figure B2, Figure B3, Figure B4, Figure B5, Figure B6, Figure B7 and Figure B8.

#### 4.3.2. Results at the National Level

The last row of Table 3 indicates that, efficiency deterioration led to an annual 1.2% increase in IWEI at the average national level, and that technical advance promoted the reduction in IWEI, with an average annual contribution of −6.4% at the average national level. Furthermore, KWE led to an annual −5.3% decrease in IWEI at the average national level, while LWE led to an annual −1.8% decrease in IWEI at the average national level. PE and FSE both contributed positively to the decrease in IWEI at the national level. By comparing these two factors, it can clearly be seen that FSE makes a larger contribution, with an average annual contribution of −7% at the average national level while the average annual contribution of PE is −5.2%. 

**Figure 4:**
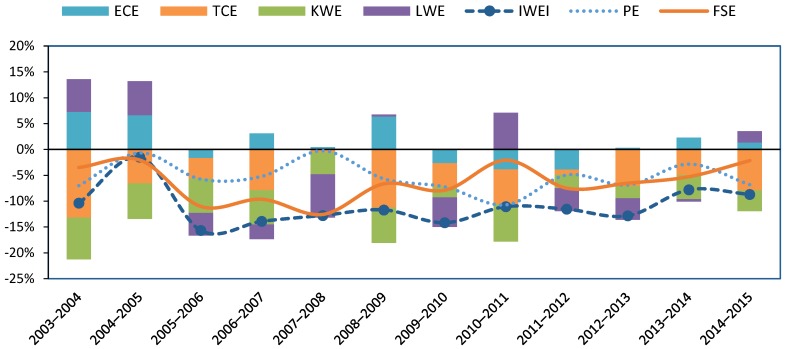
Driving factors of IWEI change at the national level, 2003–2015. ECE: the effect of technical efficiency change; TCE: the effect of technological change; KWE: the effect of capital–wastewater substitution; LWE: the effect of labor–wastewater substitution; IWEI: industrial wastewater emission intensity; PE: productivity effect; FSE: factor substitution effect.

Among the four components ECE, TCE, KWE, and LWE, it is clear that TCE is the major driving force behind the reduction in IWEI, followed by KWE. As can be seen in Figure 4, TCE and KWE were the two forces that continually drove down China’s IWEI during 2003–2015, while the other two forces, ECE and LWE, were not stable. This result is in accordance with previous research. According to Li et al. [38], technological progress is the major driving force behind the green TFP growth in Chinese industrial sectors. Chen et al. [14] found that efficiency change brings about an unstable effect in the reduction of wastewater. Additionally, Zheng et al. [58] reported that the development of industrial wastewater technology, especially membrane technology for end-of-pipe treatment, had significantly improved in China.

## 5. Discussion

### 5.1. Regional Difference in Factors Driving IWEI Change

**Figure 5:**
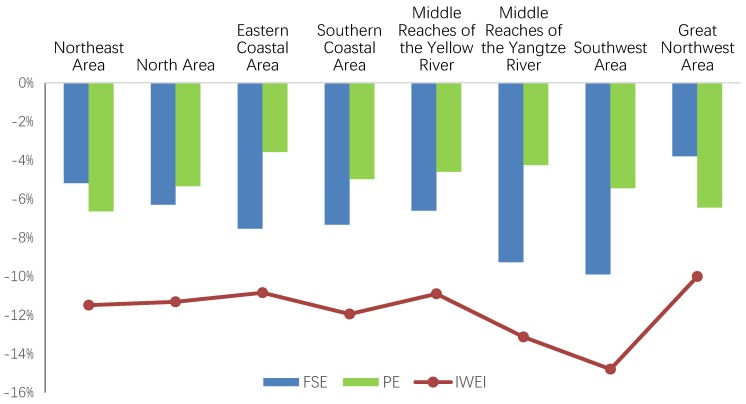
Regional differences in the driving factors of changes in industrial wastewater emission intensity, 2003–2015. PE: productivity effect; FSE: factor substitution effect; IWEI: industrial wastewater emission intensity.

Within the eight areas, there were significant differences in the driving factors of the changes in IWEI. As can be seen in Figure 5, the regional difference in FSE is much bigger than PE. Furthermore, when FSE and PE are compared, FSE contributes more to IWEI reduction in six of the eight areas; exceptions to this observation are the Northeast area and the Great Northwest area. It is also noteworthy that FSE only played a limited role in the reduction of the IWEI in the above two areas, especially in the Great Northwest area. As a result, despite the Great Northwest area achieving significant productivity growth, its IWEI still experienced the smallest decrease compared with that of the other areas during the study period. Moreover, the third and fourth lowest FSE are seen in the Middle Reaches of the Yellow River and the North area, respectively.

**Figure 6:**
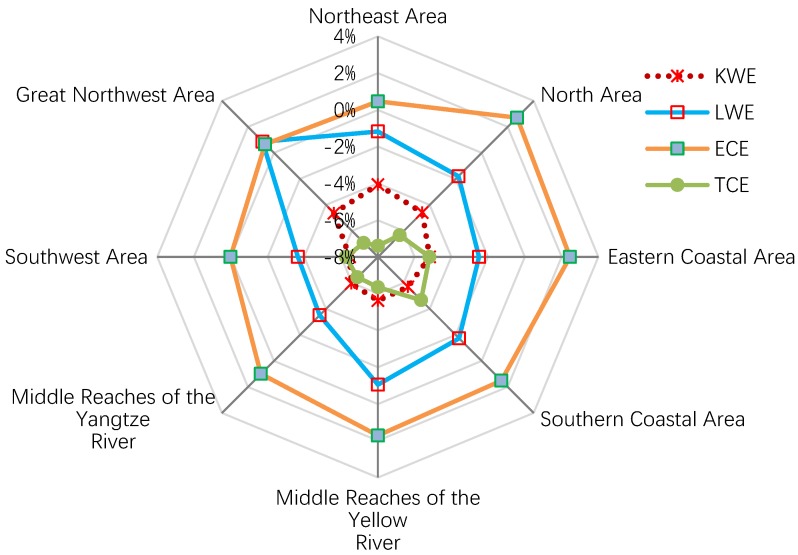
Differences in the driving factors of IWEI change in the eight areas, 2003–2015. ECE: the effect of technical efficiency change; TCE: the effect of technological change; KWE: the effect of capital–wastewater substitution; LWE: the effect of labor–wastewater substitution; IWEI: industrial wastewater emission intensity.

Further analysis reveals that the reasons for the poor performance of FSE in the four above-mentioned areas are almost the same. That is, not only was the LWE weak in these areas, but also, KWE played a much more limited role in the decline in their IWEI compared to other areas, as shown in Figure 6. For the Great Northwest area, LWE even played a negative role in the decline of its IWEI. In contrast to the KWE and LWE, the TCE contributed much more to the reduction in the IWEI in the four above-mentioned areas than in the other areas. Besides, since the difference in ECE across the eight areas is relatively slight, efficiency change and technical change cannot be the key reason for the relatively poor performance of IWEI reduction in the four above-mentioned areas, as compared with other areas. 

Based on the above analysis, we can safely conclude that compared to other areas, the shortage of KWE and LWE has impeded IWEI reduction in the Great Northwest area, the Middle Reaches of the Yellow River, the Northeast area and the North area. However, what caused the shortage of FSE in these four areas? Was it due to a lack of capital investment?

**Figure 7:**
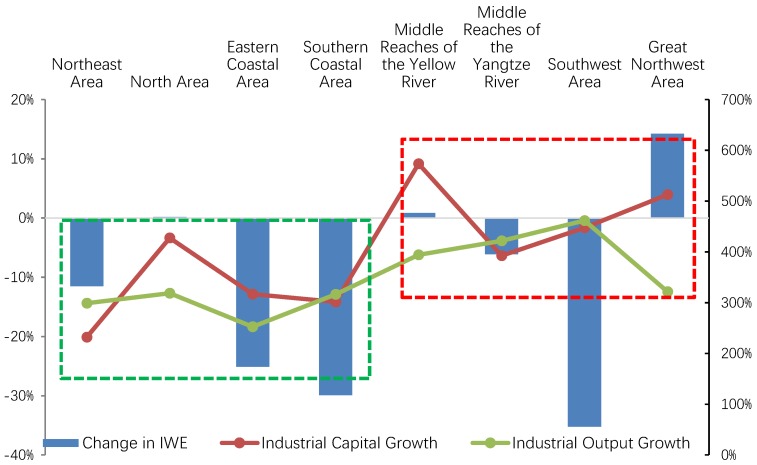
Changes in industrial capital and industrial wastewater emission in the eight areas, 2003–2015. IWE: industrial wastewater emission. Data resources: *Annual Statistic Report on Environment in China* [16] and *China Statistical Yearbook* [55].

To answer these questions, we calculated and compared the growth rate of industrial capital in the eight areas during the study periods, as shown in Figure 7. It can be clearly seen that in fact, the Middle Reaches of the Yellow River experienced the highest growth rate of industrial capital, followed by the Great Northwest area. Furthermore, the growth rate of industrial capital in the North area is also above the average level; the Northeast area experienced the lowest growth rate. Therefore, capital shortage cannot explain the insufficient KWE in the Middle Reaches of the Yellow River, the Great Northwest area and the North area at all.

Thus, the shortage of KWE in these three areas can only be explained as follows. When facing the trade-off between environmental quality and economic growth, the latter is preferred, and the three above-mentioned areas appear reluctant to substitute capital and labor for pollution. 

These results are in line with previous studies. According to Fujii et al. [39], the central and western regions have a trade-off relationship between economic and environmental performance. Furthermore, Wu et al. [59] found that the average water pollutant levels of new polluting firms are higher in the western region than in the coastal region, especially in the northwest region. Their research suggests that in the past, new polluting firms have tended to be established in the western region. This location choice by new polluting firms may result in the transfer of water pollution from the coastal to the western region of China. 

### 5.2. Envionmental Risk in Certain Areas

The above problems pose a serious challenge to the water environment in certain areas. Up to now, industrial transfer, which means that the coastal provinces transfer their outdated industries to the less developed provinces, plays an important role in China’s ongoing economic restructuring process, and further promotes industrialization in the central and western regions. However, the fact that environmental mandates vary greatly across Chinese regions raises concerns about pollution transfer [60], which could impede China’s effort to curb water pollution.

**Figure 8:**
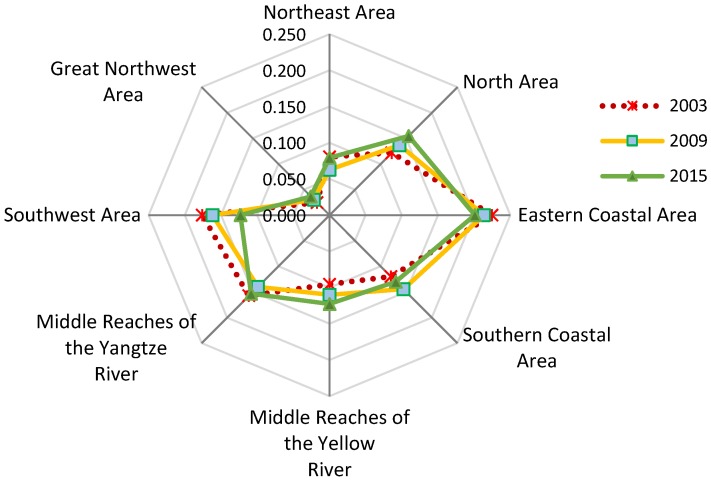
National shares of industrial wastewater emission in the eight areas, 2003–2015. Data resources: *Annual Statistic Report on Environment in China* [16].

As Figure 7 indicates, during the study periods, industrial production concentrated more quickly in mid-west China than in east China because the four areas on the right, which are located in mid-west China, experienced a significantly higher rate of industrial capital growth and output than the four areas on the left, which are located in east China. Rapid industrial concentration was accompanied by a low willingness to invest enough capital for pollution control, resulting in an undesirable outcome: the industrial wastewater emission increased in three areas, i.e., the Great Northwest area, the Middle Reaches of the Yellow River, and the North area, especially in the Great Northwest area, where it increased by about 14%. Since industrial wastewater emission decreased significantly in other areas, the three regions’ shares in the national total IWE increased dramatically during the study periods (see Figure 8).

The “Pollution Haven Hypothesis” suggests that pollution can transfer between regions with different environmental mandates within a country [61,62,63,64,65,66,67]. Additionally, according to theoretical modeling, when the income level is low, the marginal cost of pollution can be less than the marginal benefit of income increasing. Thus, the representative household’s utility would be maximized by lower pollution control standards [68]. Therefore, the willingness of poor regions to implement strict environmental regulation is usually less than that of rich regions. Furthermore, when facing a tradeoff between economic growth and environmental protection, local governments tend not to enforce the environmental policy of the central government in order to achieve better economic performance [69,70]. Consequently, depending on the potential strategic behavior of local governments and polluting firms, polluting activities may be relocated from stringent areas to lax ones [71]. Given that poverty remains the primary issue to be solved in the majority of the less developed areas in central and western China, the transfer of outdated industries from coastal provinces to them may lead to the further deterioration, or insufficient improvement of their aquatic environment.

## 6. Conclusions and Policy Implications

### 6.1. Conclusions

Considering the severe problem of water pollution and accelerating industrialization in mid-west China, a comprehensive study of the factors driving the changes in IWEI across provinces in China is relevant to future sustainable development. This study proposed a SSBM-GMI approach to decompose the change in IWEI into ECE, TCE, KWE and LWE, and performed an empirical study using Chinese provincial data from 2003 to 2015. The main conclusions can be summarized as follows. 

Firstly, technological advance was found to be the dominant driving force behind the reduction in IWEIs with an average annual contribution of −6.4% at the national level, followed by capital–wastewater substitution (−5.3%) and labor–wastewater substitution (−1.8%); technical efficiency deterioration led to an average annual 1.2% increase in IWEI at the national level. 

Secondly, there exist significant differences in the driving factors behind the reduction of IWEI across regions. The reduction in IWEIs in the Northeast area and the Great Northwest area was mainly driven by productivity growth, while the reduction of IWEIs in the other areas was mainly driven by factor substitution. Among the four components ECE, TCE, KWE, and LWE, TCE contributed the most to IWEI reduction in six of the eight areas, and KWE contributed the most to IWEI reduction in the remaining two areas, the Southwest area and the Middle Reaches of the Yangtze River, suggesting that the reduction in IWEIs in these two areas is strongly driven by capital–wastewater substitution.

Thirdly, the shortage of KWE and LWE has impeded IWEI reduction in the Great Northwest area, the Middle Reaches of the Yellow River, the Northeast area and the North area. The shortage of KWE cannot be attributed to the shortage of capital in the Great Northwest area, the Middle Reaches of the Yellow River and the North area, since these regions experienced a significantly higher rate of growth in industrial capital than other areas. This finding implies that the willingness to substitute capital for pollution in the Great Northwest area, the Middle Reaches of the Yellow River and the North area was less than that of other areas, which gives rise to vulnerability from pollution transfer, and consequently, poses a serious challenge to the water environment in these areas.

### 6.2. Policy Implications

Some policy implications can be drawn from the findings presented above with regard to further reduction of industrial wastewater in China.

First, the government should maximize the mitigating effect of “catch up” by promoting the application of cleaner production and pollution mitigation technologies nationally. The technical efficiency deterioration, which led to an average annual increase of 1.2% in IWEI at the national level, must be prevented and reversed quickly. Currently, industrial enterprises’ poor environmental awareness, outdated wastewater control equipment and procedures is one of the main reasons for high industrial wastewater emission in China [36]. The government should strictly regulate and monitor wastewater emissions, and strengthen the law enforcement on violations. By combining strict supervision and appropriate incentives, the government could effectively prompt industrial enterprises to update their wastewater mitigation technology.

Second, China should maximize the mitigating effect of factor substitutions by optimizing environmental taxes. Since cost-benefit tradeoff is the best motivation for the changes in the mode of production, China should gradually raise the relative cost of wastewater emission using environmental tax reforms to promote the substitution effect of capital and labor on wastewater emission. Most importantly, the cost of wastewater emission should be appropriately leveled in certain areas, especially in the Great Northwest area, the Middle Reaches of the Yellow River and the North area.

Third, China should prevent potential inter-regional pollution transfer by adopting a well-designed collaborative framework of environmental governance. Our findings suggest that the willingness to substitute capital for pollution in the Great Northwest area, the Middle Reaches of the Yellow River and the North area was less than that of other areas, which gives rise to vulnerability from pollution transfer. Considering the strong evidence of strategic polluting across provincial borders [72], the industrial wastewater emission increase in certain areas, especially in the Great Northwest area, which is located at the source of the main rivers in China, could lead to a further deterioration in environmental quality at the national level since the topography declines from the west to the east of China [59]. To achieve sustainable development, both government-guided arrangements, e.g., cross-regional cooperation of environmental governance, and market-driven instruments, e.g., emission trading system, should be combined to provide adequate incentives for local governments to implement stringent environmental regulation and effective environmental protection.

This study inevitably has some limitations. The nonparametric frontier approach, which does not take statistical noises into consideration was used to estimate the non-radial distance functions in this paper. In addition, industrial structure, which is truly a significant factor worthy of consideration was not included in our decomposition analysis. In future studies, parametric frontier approaches could be adopted to include statistical noises. Also, future research should investigate the factors driving the changes in industrial water pollution by combining regional and sectorial characteristics with other detailed factors in order to support more specific policy implications.

## Figures and Tables

**Table 1:** Descriptive statistics for the relevant variables.

Variable	Units	Mean	Stander. Deviation.	Max	Min	Opservations
*K*	100 million RMB	8023.56	7556.17	45,276.12	57.74	403
*L*	10 Thousand People	271.61	306.62	1568.00	1.63	403
*W*	10 Thousand Mt	73,052.02	63,715.72	296,318.00	363.00	403
*Y*	100 million RMB	4479.25	4630.30	24,092.77	17.24	403

**Table 2:** Regional distribution of changes in IWEI from 2003 to 2015. Unit: (Mt/10,000 RMB).

Province	2003	2015	Variation (%)	Province	2003	2015	Variation (%)
Beijing	13.23	3.22	−75.68	Hubei	38.42	6.34	−83.50
Tianjin	19.51	2.87	−85.29	Hunan	76.05	9.32	−87.75
Hebei	35.12	8.40	−76.07	Guangdong	25.02	7.08	−71.69
Shanxi	29.89	11.56	−61.32	Guangxi	144.29	12.99	−91.00
Inner Mongolia	31.23	5.84	−81.30	Hainan	74.93	14.83	−80.21
Liaoning	33.03	7.15	−78.34	Chongqing	107.70	7.38	−93.15
Jilin	34.27	8.73	−74.51	Sichuan	64.10	6.45	−89.94
Heilongjiang	22.96	5.14	−77.64	Guizhou	39.00	14.43	−62.99
Shanghai	22.14	6.61	−70.15	Yunnan	39.60	11.57	−70.77
Jiangsu	43.49	8.67	−80.06	Tibet	35.49	4.18	−88.23
Zhejiang	40.89	11.01	−73.07	Shaanxi	42.94	9.40	−78.11
Anhui	42.77	8.69	−79.69	Gansu	47.15	10.43	−77.88
Fujian	45.14	8.25	−81.72	Qinghai	28.52	14.17	−50.31
Jiangxi	57.15	16.13	−71.77	Ningxia	78.61	24.74	−68.52
Shandong	21.09	7.74	−63.31	Xinjiang	30.02	13.20	−56.05
Henan	38.40	9.48	−75.31	Mean	45.23	9.55	−75.98

Note: all numbers are calculated by using the 2001 constant price.

**Table 3:** Decomposition results of factors affecting IWEI change.

Area	Province	IWEI Change (1)	ECE (2)	TCE (3)	PE (4)	KWE (5)	LWE (6)	FSE (7)
Northeast Area	Liaoning	0.880	0.995	0.936	0.931	0.958	0.986	0.945
	Jilin	0.892	1.003	0.935	0.937	0.960	0.991	0.952
	Heilongjiang	0.883	1.016	0.917	0.932	0.960	0.987	0.947
	Mean	0.885	1.005	0.929	0.933	0.959	0.988	0.948
North Area	Beijing	0.889	1.058	0.898	0.950	0.961	0.974	0.936
	Tianjin	0.852	0.979	0.920	0.901	0.969	0.977	0.947
	Hebei	0.888	1.025	0.946	0.969	0.937	0.978	0.916
	Shandong	0.920	1.049	0.923	0.968	0.950	1.000	0.950
	Mean	0.887	1.027	0.922	0.947	0.954	0.982	0.937
Eastern Coastal Area	Shanghai	0.904	1.024	0.926	0.949	0.969	0.983	0.953
	Jiangsu	0.874	1.022	0.949	0.969	0.936	0.963	0.902
	Zhejiang	0.896	1.028	0.948	0.975	0.939	0.979	0.919
	Mean	0.891	1.025	0.941	0.964	0.948	0.975	0.924
Southern Coastal Area	Fujian	0.868	1.033	0.926	0.956	0.935	0.971	0.907
	Guangdong	0.900	1.021	0.940	0.960	0.959	0.978	0.937
	Hainan	0.874	0.991	0.942	0.934	0.936	0.999	0.936
	Mean	0.881	1.015	0.936	0.950	0.943	0.983	0.927
Middle Reaches of the Yellow River	Shanxi	0.924	1.038	0.932	0.967	0.945	1.010	0.955
	Inner Mongolia	0.870	0.991	0.934	0.926	0.946	0.993	0.940
	Henan	0.890	1.031	0.950	0.979	0.935	0.972	0.909
	Shaanxi	0.881	1.010	0.936	0.945	0.949	0.983	0.933
	Mean	0.891	1.017	0.938	0.954	0.944	0.989	0.934
Middle Reaches of the Yangtze River	Anhui	0.876	1.015	0.947	0.962	0.939	0.969	0.910
	Jiangxi	0.900	1.032	0.951	0.981	0.940	0.976	0.917
	Hubei	0.861	0.991	0.939	0.931	0.959	0.964	0.924
	Hunan	0.839	1.001	0.954	0.956	0.924	0.950	0.878
	Mean	0.869	1.010	0.948	0.957	0.940	0.965	0.907
Southwest Area	Guangxi	0.818	0.991	0.955	0.946	0.921	0.939	0.865
	Chongqing	0.800	0.985	0.954	0.940	0.916	0.930	0.851
	Sichuan	0.826	0.982	0.947	0.930	0.935	0.949	0.888
	Guizhou	0.921	1.027	0.935	0.961	0.953	1.005	0.958
	Yunnan	0.903	1.017	0.936	0.952	0.952	0.996	0.948
	Mean	0.852	1.000	0.945	0.946	0.935	0.963	0.901
Great Northwest Area	Tibet	0.837	0.932	0.927	0.863	0.964	1.005	0.969
	Gansu	0.882	0.996	0.936	0.932	0.941	1.005	0.946
	Qinghai	0.943	1.033	0.916	0.946	0.965	1.033	0.997
	Ningxia	0.908	1.028	0.941	0.967	0.935	1.003	0.939
	Xinjiang	0.934	1.050	0.926	0.972	0.963	0.997	0.960
	Mean	0.900	1.007	0.929	0.935	0.954	1.009	0.962
Mean value across the Country		0.881	1.012	0.936	0.948	0.947	0.982	0.930

Notes: the numbers listed above are annual mean values. IWEI: industrial wastewater emission intensity; ECE: the effect of technical efficiency change; TCE: the effect of technological change; PE: productivity effect; KWE: the effect of capital–wastewater substitution; LWE: the effect of labor–wastewater substitution; FSE: factor substitution effect.

## Appendix A

Two approaches have been generally used to integrate pollutants into the total factor framework: the first considers pollutants as an input, while the second considers pollutants as undesired output [73,74,75]. Fare et al. [76] argued that it is inappropriate to evaluate changes in social welfare by considering pollutants as an undesired output in directional distance functions. Furthermore, another study concluded that the unbalanced treatment of desired and undesired outputs may distort the evaluation of economic performance, which would mislead policy advice [77]. Although pollutants are by-products of the production process, they essentially represent unpaid costs. A substitutional relationship exists between pollutants and other production factors. That is, a given amount of output can be obtained both through the production modes of more pollution and less investment of other factors, and of less pollution and more investment of other factors. Additionally, from the perspective of natural capital, an increase in pollutant emissions actually represents a reduction in natural capital. Consequently, considering pollutant emissions as the proxy variable of natural capital investment can better reflect the connotations of green development.

## References

[1] Yale Center for Environmental Law & Policy, Yale University, Center for International Earth Science Information Network, Columbia University 2018 Environmental Performance Index. https://epi.envirocenter.yale.edu/downloads/epi2018policymakerssummaryv01.pdf.

[2] Ebenstein A. (2012). The consequences of industrialization: Evidence from water pollution and digestive cancers in china. Rev. Econ. Stat..

[3] Zhang J. (2012). The impact of water quality on health: Evidence from the drinking water infrastructure program in rural china. J. Health Econ..

[4] The Ministry of Water Resources of the People’s Republic of China China Water Resources Bulletin 2016. http://www.mwr.gov.cn/sj/tjgb/szygb/201707/t20170711_955305.html.

[5] Zhang L., Adom P.K., An Y. (2018). Regulation-induced structural break and the long-run drivers of industrial pollution intensity in china. J. Clean. Prod..

[6] Shao W. (2010). Effectiveness of water protection policy in china: A case study of Jiaxing. Sci. Total Environ..

[7] The State Council of the People’s Republic of China The Action Plan for Prevention and Control of Water Pollution. http://www.gov.cn/zhengce/content/2015--04/16/content_9613.htm.

[8] U.S. Environmental Protection Agency (2010). National Pollutant Discharge Elimination System (NPDES) Permit Writers’ Manual.

[9] U.S. Environmental Protection Agency (2011). Exposure Factors Handbook: 2011 Edition.

[10] Alsheyab M., Kusch-Brandt S. (2018). Potential recovery assessment of the embodied resources in qatar’s wastewater. Sustainability.

[11] Lei H., Xia X., Li C., Xi B. (2012). Decomposition analysis of wastewater pollutant discharges in industrial sectors of china (2001–2009) using the lmdi i method. Int. J. Environ. Res. Public Health.

[12] Fujii H., Managi S., Kaneko S. (2013). Wastewater pollution abatement in china: A comparative study of fifteen industrial sectors from 1998 to 2010. J. Environ. Prot..

[13] Geng Y., Wang M., Sarkis J., Xue B., Zhang L., Fujita T., Yu X., Ren W., Zhang L., Dong H. (2014). Spatial-temporal patterns and driving factors for industrial wastewater emission in china. J. Clean. Prod..

[14] Chen K., Liu X., Ding L., Huang G., Li Z. (2016). Spatial characteristics and driving factors of provincial wastewater discharge in china. Int. J. Environ. Res. Public Health.

[15] Jia J., Jian H., Xie D., Gu Z., Chen C. (2017). Multi-perspectives’ comparisons and mitigating implications for the cod and nh3-n discharges into the wastewater from the industrial sector of china. Water.

[16] The Ministry of Environmental Protection of the People’s Republic of China (2006–2016). Annual Statistic Report on Environment in China.

[17] Grossman G.M., Krueger A.B. (1991). Environmental Impacts of a North American Free Trade Agreement.

[18] Panayotou T. (1993). Empirical Tests and Policy Analysis of Environmental Degradation at Different Stages of Economic Development.

[19] Grossman G.M., Krueger A.B. (1995). Economic growth and the environment. Q. J. Econ..

[20] Hamilton C., Turton H. (2002). Determinants of emissions growth in OECD countries. Energy Policy.

[21] Stern D.I. (2002). Explaining changes in global sulfur emissions: An econometric decomposition approach. Ecol. Econ..

[22] Bruvoll A., Medin H. (2003). Factors behind the environmental kuznets curve. A decomposition of the changes in air pollution. Environ. Resour. Econ..

[23] Su W., Zhang C. (2011). The inspection of EKC hypothesis in China based on heterogeneity. Stat. Res..

[24] Fan S., Gao T. (2017). Change status of industrial pollution of china and its ekc empirical analysis based on ecological threshold perspective. Ecol. Econ..

[25] Guo W.-W. (2018). EKC Analysis of Three Industrial Wastes of Five Provinces in Northwest China.

[26] Ang B.W. (2005). The LMDI approach to decomposition analysis: A practical guide. Energy Policy.

[27] Shao C., Guan Y., Wan Z., Guo C., Chu C., Ju M. (2014). Performance and decomposition analyses of carbon emissions from industrial energy consumption in Tianjin, China. J. Clean. Prod..

[28] Li Y., Luo Y., Wang Y., Wang L., Shen M. (2017). Decomposing the decoupling of water consumption and economic growth in china’s textile industry. Sustainability.

[29] Lin B., Du K. (2014). Decomposing energy intensity change: A combination of index decomposition analysis and production-theoretical decomposition analysis. Appl. Energy.

[30] Du K., Lin B. (2015). Understanding the rapid growth of china’s energy consumption: A comprehensive decomposition framework. Energy.

[31] Li A.J., Zhang A.Z., Zhou Y.X., Yao X. (2017). Decomposition analysis of factors affecting carbon dioxide emissions across provinces in china. J. Clean. Prod..

[32] Wang Q., Zhang C., Cai W. (2017). Factor substitution and energy productivity fluctuation in china: A parametric decomposition analysis. Energy Policy.

[33] Chen X., Fan D. (2009). Empirical Study on Status of Industrial Water Pollution and Treatment Efficiency in China. Stat. Inf. Forum.

[34] Sala-Garrido R., Molinos-Senante M., Hernández-Sancho F. (2011). Comparing the efficiency of wastewater treatment technologies through a dea metafrontier model. Chem. Eng. J..

[35] Liu Y., Gong B., Liu X. (2017). An appraise of wastewater treatment efficiency in china mineral industries based on dea models with undesirable outputs. Chin. J. Environ. Eng..

[36] Yang W., Li L. (2017). Efficiency evaluation and policy analysis of industrial wastewater control in China. Energies.

[37] Fujii H., Managi S. (2017). Wastewater management efficiency and determinant factors in the Chinese industrial sector from 2004 to 2014. Water.

[38] Li H., Zhang J., Osei E., Yu M. (2018). Sustainable development of china’s industrial economy: An empirical study of the period 2001–2011. Sustainability.

[39] Fujii H., Cao J., Managi S. (2015). Decomposition of productivity considering multi-environmental pollutants in Chinese industrial sector. Rev. Dev. Econ..

[40] Chen C., Lan Q., Gao M., Sun Y. (2018). Green total factor productivity growth and its determinants in China’s industrial economy. Sustainability.

[41] Fare R., Grosskopf S., Pasurkajr C. (2007). Environmental production functions and environmental directional distance functions. Energy.

[42] Zhou P., Ang B.W. (2008). Decomposition of aggregate co2 emissions: A production-theoretical approach. Energy Econ..

[43] Zhang N., Zhou P., Choi Y. (2013). Energy efficiency, co2 emission performance and technology gaps in fossil fuel electricity generation in Korea: A meta-frontier non-radial directional distance function analysis. Energy Policy.

[44] Timmer M.P., Los B. (2005). Localized innovation and productivity growth in asia: An intertemporal dea approach. J. Prod. Anal..

[45] Tone K. (2001). A slacks-based measure of efficiency in data envelopment analysis. Eur. J. Oper. Res..

[46] Tone K. (2002). A slacks-based measure of super-efficiency in data envelopment analysis. Eur. J. Oper. Res..

[47] Pastor J.T., Lovell C.A.K. (2005). A global malmquist productivity index. Econ. Lett..

[48] Oh D.-H. (2010). A global malmquist-luenberger productivity index. J. Prod. Anal..

[49] Oh D.-H., Heshmati A. (2010). A sequential malmquist–luenberger productivity index: Environmentally sensitive productivity growth considering the progressive nature of technology. Energy Econ..

[50] Wang C. (2007). Decomposing energy productivity change: A distance function approach. Energy.

[51] Wang C. (2011). Sources of energy productivity growth and its distribution dynamics in china. Resour. Energy Econ..

[52] Ang B.W. (2004). Decomposition analysis for policymaking in energy: Which is the preferred method?. Energy Policy.

[53] Boyd G.A., Pang J.X. (2000). Estimating the linkage between energy efficiency and productivity. Energy Policy.

[54] Smyth R., Narayan P.K., Shi H. (2011). Substitution between energy and classical factor inputs in the chinese steel sector. Appl. Energy.

[55] National Bureau of Statistics of the People’s Republic of China (2004–2016). China Statistical Yearbook.

[56] Ministry of Environmental Protection of the People’s Republic of China (2004–2006). China’s Environmental Yearbook.

[57] Hall R.E., Jones C.I. (1999). Why do some countries produce so much more output per worker than others?. Q. J. Econ..

[58] Zheng X., Zhang Z., Yu D., Chen X., Cheng R., Min S., Wang J., Xiao Q., Wang J. (2015). Overview of membrane technology applications for industrial wastewater treatment in China to increase water supply. Resour. Conserv. Recycl..

[59] Wu H., Guo H., Zhang B., Bu M. (2017). Westward movement of new polluting firms in China: Pollution reduction mandates and location choice. J. Comp. Econ..

[60] Dean J.M., Lovely M.E., Wang H. (2005). Are Foreign Investors Attracted to Weak Environmental Regulations? Evaluating the Evidence from China.

[61] Copeland B.R., Taylor M.S. (1999). Trade, spatial separation, and the environment. J. Int. Econ..

[62] Antweiler W., Copeland R., Taylor M.S. (2001). Is free trade good for the emissions: 1950–2050. Rev. Econ. Stat..

[63] Copeland B.R., Taylor M.S. (2004). Trade, Tragedy, and the Commons.

[64] Kahn M. (2004). Domestic pollution havens: Evidence from cancer deaths in border counties. J. Urban Econ..

[65] Kellenberg D.K. (2009). An empirical investigation of the pollution haven effect with strategic environment and trade policy. J. Int. Econ..

[66] Wagner U.J., Timmins C.D. (2009). Agglomeration effects in foreign direct investment and the pollution haven hypothesis. Environ. Resour. Econ..

[67] Chung S. (2014). Environmental regulation and foreign direct investment: Evidence from south Korea. J. Dev. Econ..

[68] Stokey N.L. (1998). Are there limits to growth?. Int. Econ. Rev..

[69] Chow G.C. (2010). China’s Environmental Policy: A Critical Survey.

[70] Zhang D., Aunan K., Seip H.M., Vennemo H. (2011). The energy intensity target in China’s 11th five-year plan period—Local implementation and achievements in Shanxi province. Energy Policy.

[71] Cai H., Chen Y., Gong Q. (2016). Polluting thy neighbor: Unintended consequences of China’s pollution reduction mandates. J. Environ. Econ. Manag..

[72] Shen M., Yang Y. (2017). The water pollution policy regime shift and boundary pollution: Evidence from the change of water pollution levels in China. Sustainability.

[73] Hailu A., Veeman T.S. (2001). Non-parametric productivity analysis with undesirable outputs: An application to the canadian pulp and paper industry. Am. J. Agric. Econ..

[74] Korhonen P.J., Luptacik M. (2004). Eco-efficiency analysis of power plants: An extension of data envelopment analysis. Eur. J. Oper. Res..

[75] Kumar S. (2006). Environmentally sensitive productivity growth: A global analysis using malmquist–luenberger index. Ecol. Econ..

[76] Färe R., Grosskopf S., Pasurka J., Carl A. (2001). Accounting for air pollution emissions in measures of state manufacturing productivity growth. J. Reg. Sci..

[77] Hailu A., Veeman T.S. (2000). Environmentally sensitive productivity analysis of the canadian pulp and paper industry, 1959–1994: An input distance function approach. J. Environ. Econ. Manag..

